# The membrane tethered transcription factor *EcbZIP17* from finger millet promotes plant growth and enhances tolerance to abiotic stresses

**DOI:** 10.1038/s41598-018-19766-4

**Published:** 2018-02-01

**Authors:** Chopperla Ramakrishna, Sonam Singh, Sangala Raghavendrarao, Jasdeep C. Padaria, Sasmita Mohanty, Tilak Raj Sharma, Amolkumar U. Solanke

**Affiliations:** 1 ICAR-National Research Centre on Plant Biotechnology, Pusa Campus, New Delhi, 110012 India; 2 School of Biotechnology, KIIT University, Bhubaneswar, Odisha 751024 India

## Abstract

The occurrence of various stresses, as the outcome of global climate change, results in the yield losses of crop plants. Prospecting of genes in stress tolerant plant species may help to protect and improve their agronomic performance. Finger millet (*Eleusine coracana* L.) is a valuable source of superior genes and alleles for stress tolerance. In this study, we isolated a novel endoplasmic reticulum (ER) membrane tethered bZIP transcription factor from finger millet, *EcbZIP17*. Transgenic tobacco plants overexpressing this gene showed better vegetative growth and seed yield compared with wild type (WT) plants under optimal growth conditions and confirmed upregulation of brassinosteroid signalling genes. Under various abiotic stresses, such as 250 mM NaCl, 10% PEG6000, 400 mM mannitol, water withdrawal, and heat stress, the transgenic plants showed higher germination rate, biomass, primary and secondary root formation, and recovery rate, compared with WT plants. The transgenic plants exposed to an ER stress inducer resulted in greater leaf diameter and plant height as well as higher expression of the ER stress-responsive genes *BiP*, *PDIL*, and *CRT1*. Overall, our results indicated that *EcbZIP17* improves plant growth at optimal conditions through brassinosteroid signalling and provide tolerance to various environmental stresses via ER signalling pathways.

## Introduction

Environmental stresses, such as heat, drought, cold, and salt, affect crop plants, causing yield losses up to 50%^1,2^. In the past two decades, numerous abiotic stress responsive genes have been isolated and characterised from diverse plant species^3,4^. Among all plant species, finger millet (*Eleusine coracana* L.) is one of the richest sources of abiotic stress tolerant genes, and this crop is well adapted to a wide range of environments such as drought, salt, water logging, and a few diseases^5^. Therefore, as a ‘climate resilient crop’, finger millet is considered an attractive system for functional genomics studies^6–8^. It is widely grown in the arid areas of Africa, Asia, and southern India, which account for approximately 12% of the global millet production area^9^. Finger millet is rich in calcium, fibre, iron and hence, is recognised as a ‘nutri-cereal’ or ‘super cereal’. It is an allotetraploid with AABB genome and a basic chromosome number of 9 (2n = 4x = 36).

Transcription factors (TFs) regulate the gene expression, as they interact with regulatory proteins (co-transcription factors) and bind to the *cis*-elements in the promoter regions of the target genes^10–12^. Numerous TF families, such as WRKY, ERF, bZIP, and MYB, have been identified to be involved in the stress-responsive gene regulation^13–16^. Among these, basic leucine zipper (bZIP) is one of the largest families in plants, characterised by a basic domain for sequence-specific DNA binding and a leucine zipper required to dimerise when binds to the two DNA regions, preferentially with an ACGT core^15,17–19^. Genetic, molecular, and biochemical analyses have shown that *bZIP* transcription factors are master regulators of many essential plant processes, such as pathogen defence, hormone and sugar signalling, and response to light, drought, salt, and cold stress, that along with unfolded protein responses provide tolerance to biotic and abiotic stresses^20–24^. Among plant species *Glycine max* (352) has the highest number of bZIP family members followed by *Kalanchoe marnieriana* (325), *Panicum virgatum* (324), *Gossypium raimondi* (272), *Physcomitrella patens* (265), *Brassica napus* (264), *Salix purpurea* (226) and the least number of bZIP members found in *Picea sitchensis* (17), Plant transcription factor database 4.0. (http://planttfdb.cbi.pku.edu.cn/index.php). Based on the conserved region, bZIPs in *Arabidopsis* (75) and rice (89) are subdivided into 10 different types: A to I and S in the former and A to J in the latter^17,19^. Of the 10 types, the Type-B bZIP TFs play a key role in endoplasmic reticulum (ER) stress, also known as unfolded protein response (UPR)^25,26^. UPR occurs when misfolded or unfolded proteins accumulate in the ER. Type-B bZIP proteins have a transmembrane (TM) domain and a site 1 protease (S1P) cleavage site in the luminal region^27^. Abiotic stress, such as heat, drought, and salt, affects protein folding and activates ER stress^28^. ER is the master site for the co-transcriptional translation and formation of membrane tethered transcription factors (MTTFs) such as AtbZIP17, AtbZIP28, AtbZIP49, and AtbZIP60. MTTFs are localised in the ER membrane under optimal growth conditions, whereas under environmental stress conditions or treatment with ER stress agents, such as tunicamycin (TM) or dithiothreitol (DTT), they are transported to the Golgi apparatus and proteolytically processed by the Golgi-localised proteases S1P and S2P. The processed forms of MTTFs enter the nucleus to trigger stress responsive genes such as binding protein (*BiP*), protein disulphide isomerise (*PDIL*), calnexin (*CNX*), and calreticulin (*CRT1*)^29^.

BiP is a classic marker and central regulator of the UPR when unfolded proteins aggregate in the ER^30,31^. Under normal cellular conditions, BiP is associated with the C-terminal region of signalling molecules such as bZIP17 or bZIP28^32^. Under stress conditions, the excess accumulation of unfolded proteins reduces the number of free BiP molecules in the ER, because of the higher affinity of BiP molecules with unfolded proteins than with signalling molecules^33,34^, leading to the activation of BiP dissociated signalling molecules. The latter signalling molecules translocate from ER to the nucleus through the Golgi vesicle and processed by S1P and S2P^32,35^. The processed form of signalling molecules activates ER stress responsive genes to overcome the excess accumulation of unfolded proteins in the ER. Additionally, the intra-and inter-chain disulphide bridge formation is catalysed by protein disulphide isomerase (PDI) to inhibit the aggregation of misfolded proteins in the ER^36^. Previous studies showed that mutations in the active site of yeast PDI results in DTT sensitivity and thus, reduction in the rate of protein folding^37^. Calreticulin (CRT), a high capacity Ca^2+^ binding multifunctional protein, plays a key role in the intracellular Ca^2+^ homeostasis and as a molecular chaperone, assists in glycoprotein folding^38,39^. Recent studies have revealed that plant UPR has various effects on important cellular processes such as adaptability to environmental conditions and productivity^26,40–43^. Although finger millet is a rich reservoir of genes for abiotic stress tolerance, only a limited number of them, such as *EcDehydrin7*, *EcNAC1*, *EcNAC67*, *EcbHLH57*, and *EcbZIP60*, has been characterised^44–49^. In the present study, we identified *EcbZIP17* from finger millet and characterised it for different abiotic stresses. Additionally, we generated transgenic tobacco plants overexpressing *EcbZIP17* to determine the role of the gene under optimal and various abiotic stress conditions.

## Results

### Isolation and sequence analysis of *EcbZIP17*

The partial EST sequence of *EcbZIP17* obtained from the finger millet EST library was 533 bp in length, lacking a 5′ region. Therefore, Rapid amplification of cDNA ends (5′ RACE) was carried out to obtain full-length cDNA with complete ORF. Sequencing analysis revealed that the ORF of *EcbZIP17* was 1,722 bp in length (Supplementary Fig. 1), translated into a product of 573 aa with a molecular weight of 61 kDa. EcbZIP17 was predicted as a nuclear protein with a leucine rich export signal of 75–83 aa (Supplementary Fig. 2a,b), a BRLZ domain of 120–184 aa, a TM domain of 249–271 aa, a nuclear localisation signal (NLS) of 223–245 aa, and a canonical S1P protease site of 279–282 aa (Supplementary Fig. 3a). Homology analysis of EcbZIP17 (AHZ30615.1) revealed 76% identity with a *Sorghum bicolor* hypothetical protein (Sb02g041070), 75% identity with the maize ZmbZIP17 (BT040011), 48% identity with the rice OsbZIP39 (Os05g34050), and 40% identity with the *Arabidopsis* AtbZIP17 (NM_129659.2), mainly at the BRLZ domain, TM domain, NLS, and S1P protease site, as well as similarity at the evolutionary and common motif level. However some more homologous proteins (OsbZIP60 (Os07g44950), ZmbZIP28 (BT067808), ZmbZIP60 (BT086464), AtbZIP60 (NM_103458), AtbZIP28 (NM_001202616), and AtbZIP49 (At3g56660)) were used to predict the exact evolutionary relationship in phylogenetic tree construction (Supplementary Fig. 3b–d). The homologous proteins are known to play a key role in the ER stress response or the UPR. Thus, an NLS at the N-terminal end facing the cytosol and a TM domain at the C-terminal end facing the ER lumen, which is a characteristic feature of the homologous proteins, indicated the putative localisation of EcbZIP17 in the ER membrane.

### Expression of *EcbZIP17* in finger millet

**Figure 1:**
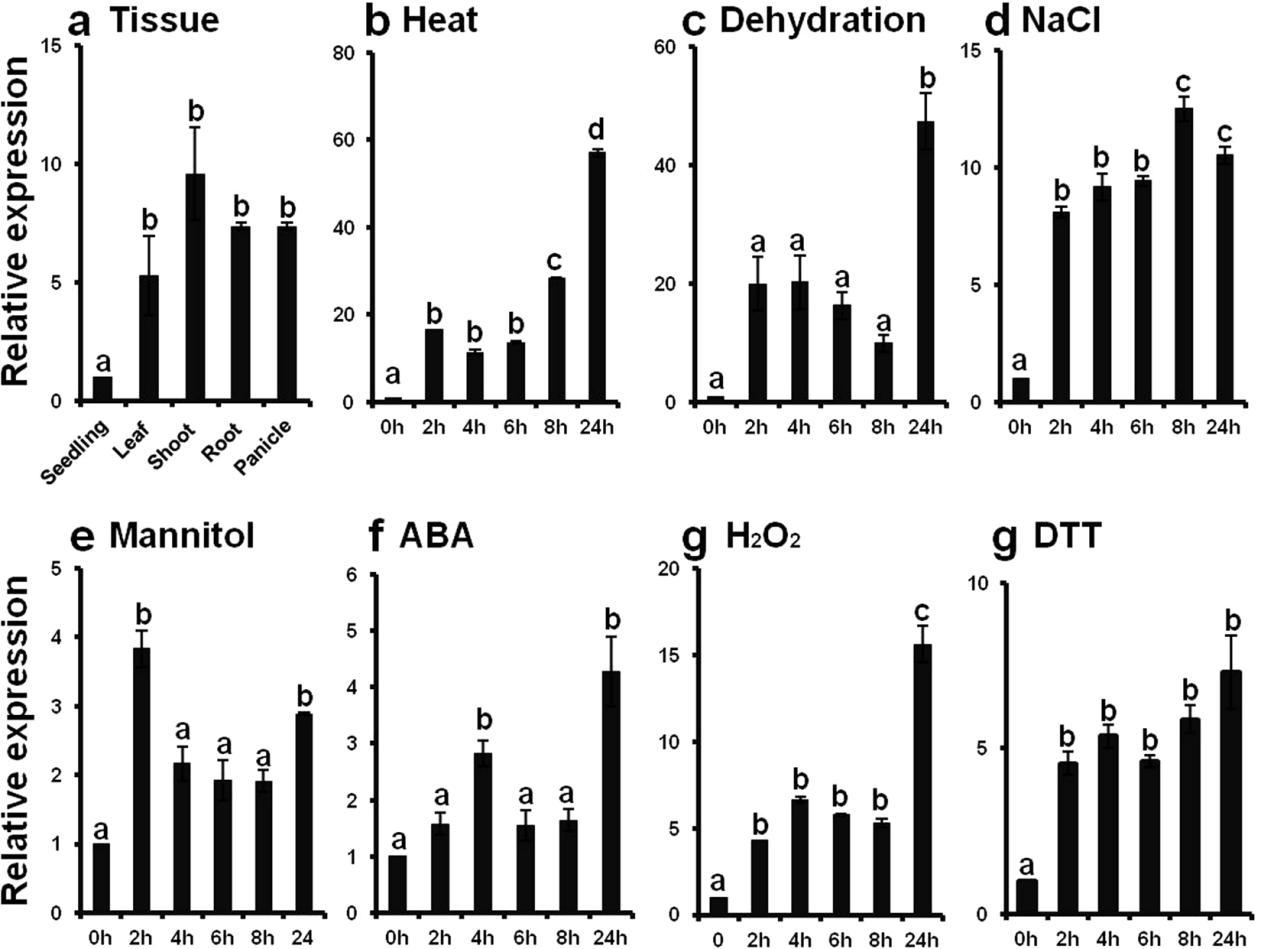
Expression pattern of the finger millet *EcbZIP17* transcript. (**a**) Expression in different plant organs; in three leaf stage seedlings treated with (**b**) Heat (42 °C), (**c**) Dehydration, (**d**) NaCl (250 mM), (**e**) Mannitol (300 mM), (**f**) ABA (100 µM), (**g**) H_2_O_2_ (25 mM) and (**h**) DTT (2.5 mM) at indicated time points respectively. β-Tubulin was used as the internal reference gene to normalize the data. Data represents mean ± SE of three technical replicates. Different letters above the error bars indicate significant difference between 0hr and indicated time periods of stress treatment, based on one-way analysis of variance (P < 0.05).

The expression of *EcbZIP17* was observed in all the vegetative parts of the plant, including the leaf, shoot, root, panicle, and germinated seedling; however, the highest expression was noted in the shoot (Fig. 1a), whereas the transcript levels were similar in the panicle and the root. The expression of *EcbZIP17* was upregulated under all stress treatments. Under heat, dehydration, ABA, and H_2_O_2_ stresses, the expression levels of *EcbZIP17* markedly increased after 24 h of treatment (Fig. 1b,c,f,g), whereas under NaCl stress, the expression level increased gradually and then, decreased after 8 h of treatment (Fig. 1d). Under mannitol treatment, the expression level initially increased and then, decreased after 2 h of treatment (Fig. 1e). Apart from the abiotic and oxidative stresses, *EcbZIP17* was also upregulated under DTT stress, and the highest expression was observed after 24 h of treatment (Fig. 1h).

### Development of transgenic tobacco plants overexpressing *EcbZIP17*

A total of 24 putative transgenic lines were generated based on the preliminary screening on kanamycin selection medium. Of these, 13 lines were confirmed by PCR using *EcbZIP17* and *npt*II specific primers (Supplementary Fig. 4a,b). Southern blot hybridisation revealed that all transgenic lines were independent events with estimated integrations, ranging from one to nine copies of T-DNA. The transgenic lines EcbZIP17E, EcbZIP17O, and EcbZIP17S showed single integrations, whereas the transgenic line EcbZIP17W showed double integrations (Supplementary Fig. 4c). The expression level of *EcbZIP17* was higher in EcbZIP17E, EcbZIP17O, EcbZIP17S, and EcbZIP17W compared with the other nine transgenic lines (Supplementary Fig. 4d). Therefore, the transgenic lines, EcbZIP17E and EcbZIP17S with a single T-DNA insertion, and EcbZIP17W with a double insertion and higher *EcbZIP17* expression levels were used for all the experiments in this study, whereas the transgenic lines, EcbZIP17Y and EcbZIP17Z were also used for mannitol stress and drought recovery experiments, respectively, in addition to the other three lines.

### Growth of *EcbZIP17* transgenic plants under optimal growth conditions

**Figure 2:**
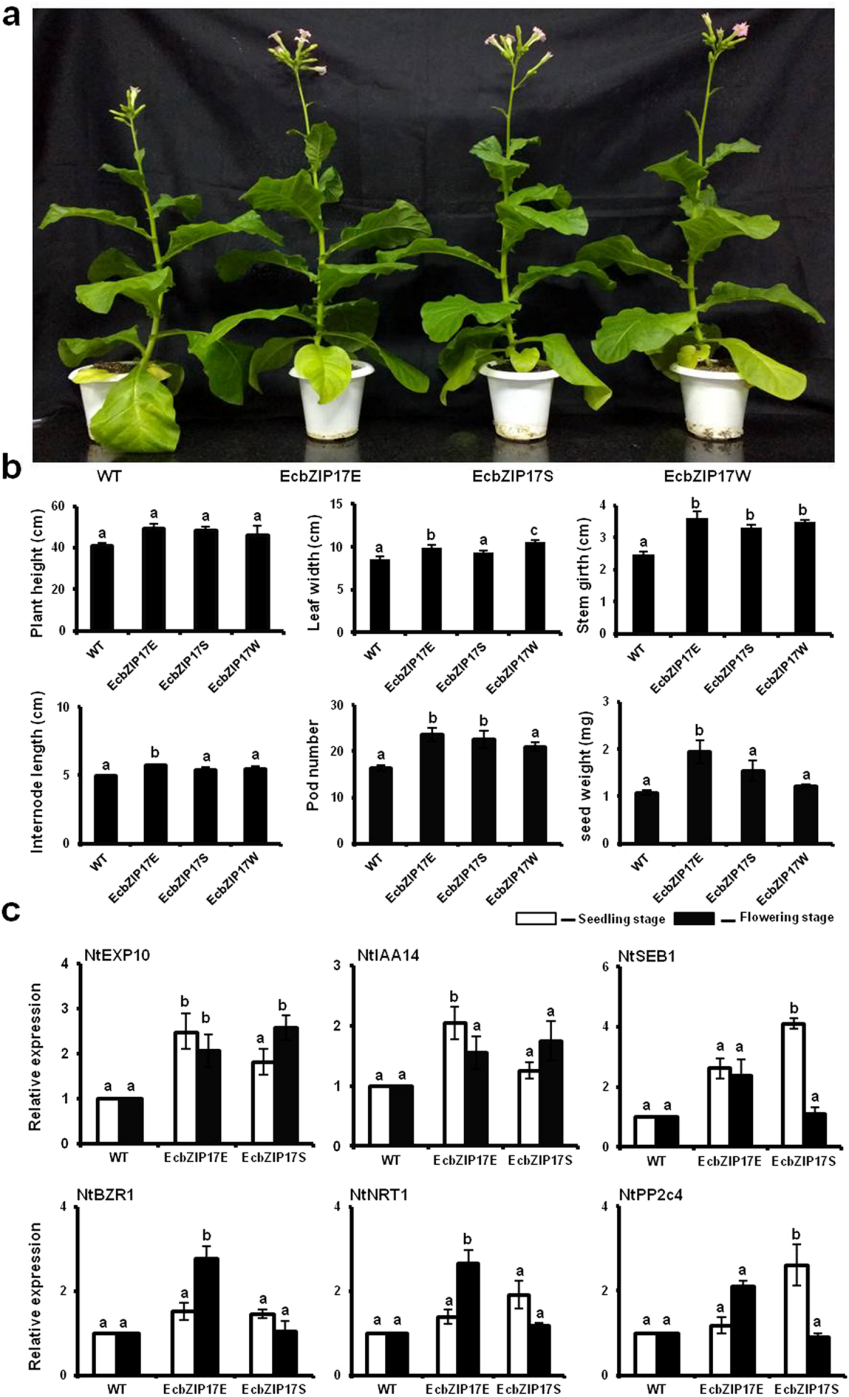
Phenotypic analysis and expression profiling of Brassinosteroid responsive genes in EcbZIP17-T1 transgenic tobacco plants. (**a**) Representative image after 115 days at normal growth condition. (**b**) Graph chart representation of plant height, leaf width, stem girth, internode length, pod number and total seed weight of WT and transgenic lines at flowering stage. (**c**) Expression pattern of Brassinosteroid responsive genes in seedling and flowering stage of WT and transgenic lines. Data represents mean ± SE of three replicates (n = 3). According to one way ANOVA (P < 0.05) different lower case letters above the error bars indicate significant difference in expression of transgenic lines over WT after normalization. L25 was used as internal reference gene for normalization.

The vegetative growth (i.e. plant height, number of leaves per plant, and leaf area) of transgenic and WT plants was almost similar until the initiation of flowering, whereas significant differences were identified during the transition from the vegetative to the reproductive stage (Fig. 2a). In transgenic plants, we observed an increase in the height by 12–20% and in the girth of the stem by 40–44% within a week. Additionally, a significant increase was observed in the size of the leaves per plant (15–23%), the internode distance (12–16%), the number of pods per plant (28–44%), and the total weight of seeds per plant (14–81%) in transgenic plants (Fig. 2b). Since the transgenic plants performed better under optimal growth conditions than the WT plants, we investigated the expression level of growth related genes of the brassinosteroid signalling pathway, including (*EXP10*, *NtIAA14, NtSEB1, NtBZR1, NtNTR1, NtPP2c4*) at the seedling and flowering stages. All these genes are showing better expression at both seedling and flowering stage in transgenic plants compared to WT (Fig. 2c).

### Tolerance of *EcbZIP17* transgenic plants to salt stress

**Figure 3:**
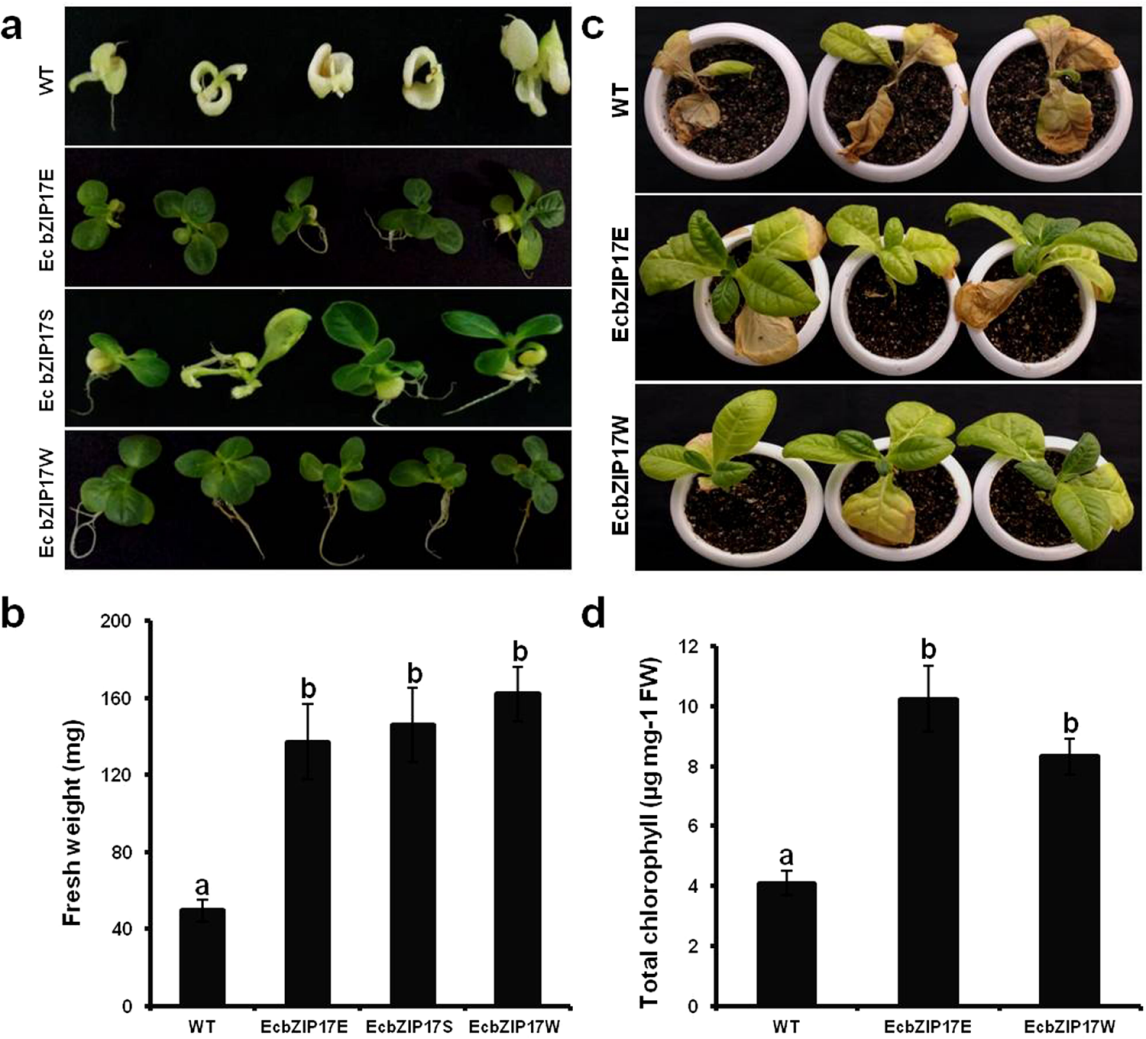
*EcbZIP17* over-expressing tobacco plants tolerant to salt stress. (**a**) Representative image of seedlings from WT and EcbZIP17-T1 transgenic lines (EcbZIP17E, EcbZIP17S, EcbZIP17W) after 30 d of germination under 250 mM NaCl stress, (**b**) Fresh weight of the same plants after 30 d of germination, (**c**) Salt stress recovery response (one month old WT and *EcbZIP17*-T1 transgenic lines were subjected to salt stress by supplementing with 250 mM NaCl solution for every alternative three days for one month time period and subjected to recovery), (**d**) Total chlorophyll content of WT and transgenic lines after NaCl stress. Data represents mean ± SE of three replicates (n = 3). According to one way ANOVA values of transgenic plants significantly different from WT and indicated by lower case letters (P < 0.05).

At germination under NaCl stress, the leaf colour of WT plants showed a severe bleaching, whereas that of all transgenic plants remained green (Fig. 3a). The appearance of transgenic plants that were non-transformants was similar to that of WT plants due to the absence of T-DNA. The fresh weight of transgenic plants was significantly higher than that of WT plants (Fig. 3b). Under salt stress experiment of 30 d old plants, the leaf architecture and chlorophyll content of transgenic and WT plants were similar before and after the stress treatment. However, after one-month of salt stress, the leaf architecture of WT plants markedly changed compared with that of transgenic plants. After 40 d of recovery from the stress treatment, the WT plants were completely bleached, whereas the transgenic plants were fully recovered (Fig. 3c). The chlorophyll content of transgenic and WT plants was lower under salt stress conditions compared with that under optimal growth conditions; however, transgenic plants showed significantly higher chlorophyll content compared with that of WT plants after 40 d of recovery (Fig. 3d). Additionally, transgenic plants also showed better MSI during stress (Supplementary Fig. 5).

### Tolerance of *EcbZIP17* transgenic plants to mannitol stress

**Figure 4:**
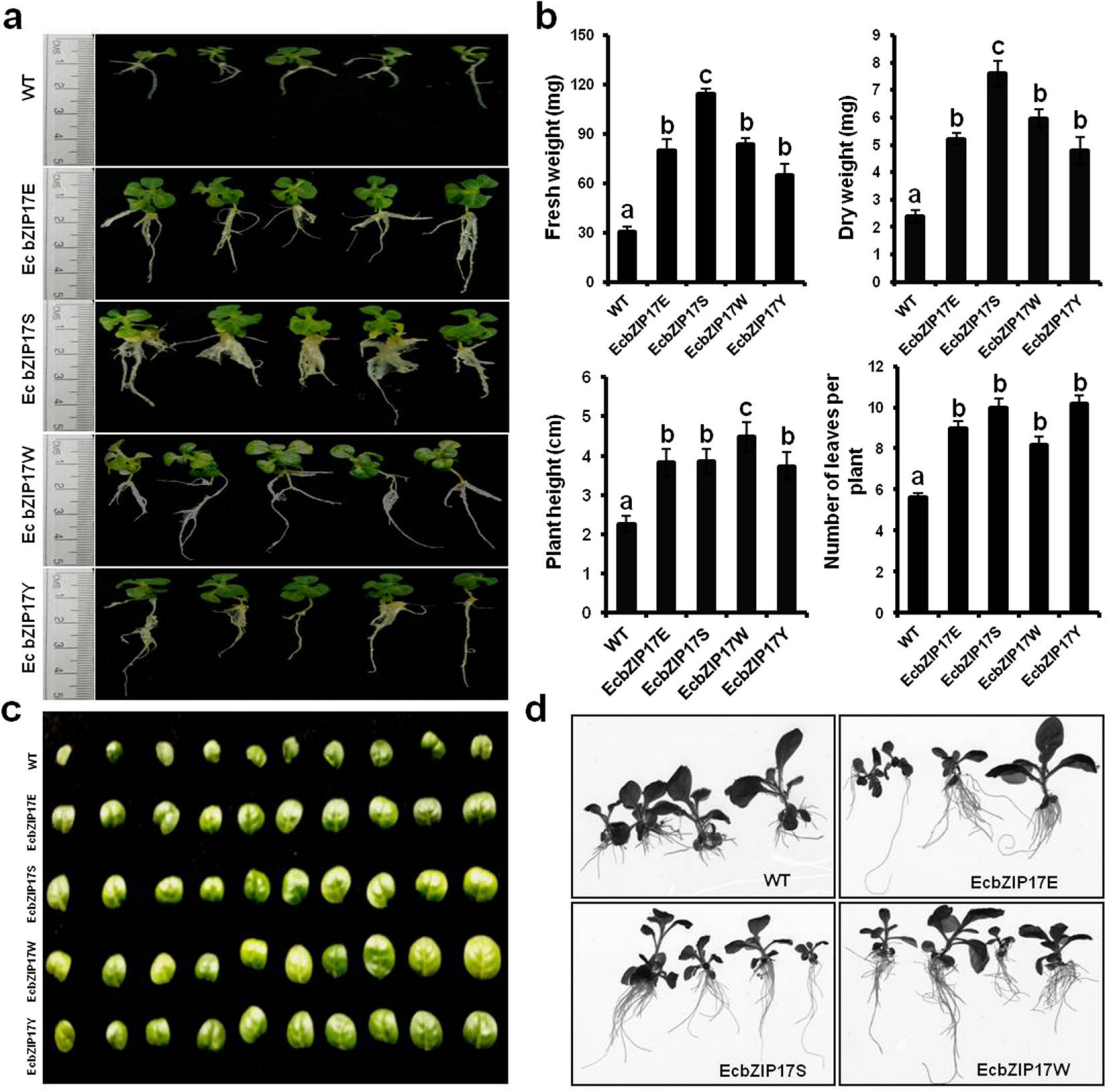
*EcbZIP17* over-expressing tobacco plants tolerant to mannitol stress. (**a**) WT and EcbZIP17-T1 transgenic lines (EcbZIP17E, EcbZIP17S, EcbZIP17W, EcbZIP17Y) were germinated on MS plain and MS medium supplemented with Kanamycin and ten days after germination, twenty seedlings from each line were transferred to MS medium supplemented with 400 mM mannitol and the representative image after 45 days. (**b**) Graph chart representation of fresh weight, dry weight, plant height and number of leaves per plant. (**c**) Representative image of randomly collected ten leaves from WT and transgenic lines. (**d**) Ten days old seedlings of WT and EcbZIP17-T1 transgenic lines (EcbZIP17E, EcbZIP17S, EcbZIP17W) were placed on magenta boxes having 250 mM mannitol hydroponic solution for one month and images were captured. Data represents mean ± SE of atleast five replicates. According to one way ANOVA, values of transgenic plants significantly different from WT plants and indicated by lower case letters above the error bars (P < 0.05).

After 45 d of mannitol stress, the primary root length, number of secondary roots, fresh weight, dry weight, number of leaves per plant, diameter of leaves, and plant height were significantly higher in transgenic plants than in WT plants (Fig. 4a–d; Supplementary Fig. 6). Compared with the WT plants, the transgenic plants showed a higher fresh weight by 114–276%, number of leaves per plant by 46–82%, and plant height by 65–98%.

### Tolerance of *EcbZIP17* transgenic plants to PEG

Under PEG 6000, the vegetative growth of transgenic plants was significantly higher than that of WT plants (Supplementary Fig. 7a). Transgenic plants showed a maximum increase of 73% in fresh weight and 76% in dry weight compared with WT plants (Supplementary Fig. 7b). However, no significant differences were observed in the chlorophyll content of transgenic and WT plants (Supplementary Fig. 8).

### Plant height and yield of *EcbZIP17* transgenic plants after drought recovery

**Figure 5:**
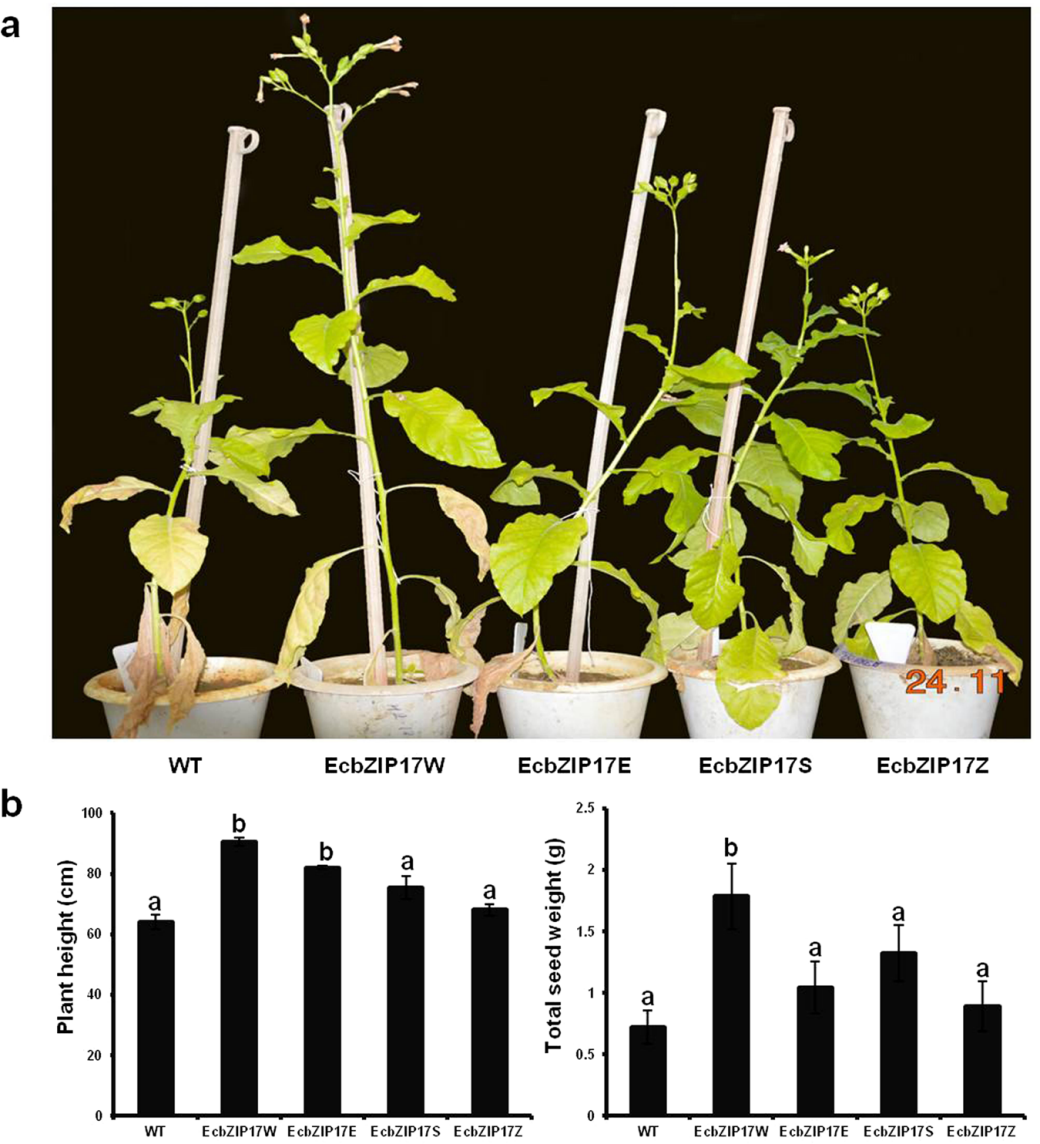
Drought recovery response of *EcbZIP17* over-expressing tobacco plants. (**a**) Representative image of the WT and EcbZIP17-T1 transgenic lines after drought recovery. Fifteen days old seedlings of WT (germinated on MS plain) and *EcbZIP17* transgenic lines EcbZIP17W, EcbZIP17E, EcbZIP17S, EcbZIP17Z (germinated on MS-Kanamycin) were placed on a tray containing soil and soil rite mixture (1:1). These plants were acclimatized for further fifteen days and subjected to drought stress by water withdrawal for eleven days and recovered. (**b**) Plant height and total seed weight of recovered plants. Data represents mean ± SE of five replicates (n = 5). According to one way ANOVA, values of transgenic plants significantly different from WT plants and indicated by lower case letters above the error bars (P < 0.05).

Wilting was noticed in both transgenic and WT plants, after 11 d of drought stress. Further, 30 d of recovery by watering, all plants were visibly recovered. However, during the flowering stage, the plant height and pod weight of transgenic plants were significantly higher than those of WT plants (Fig. 5a,b).

### Tolerance of *EcbZIP17* transgenic plants to heat stress

**Figure 6:**
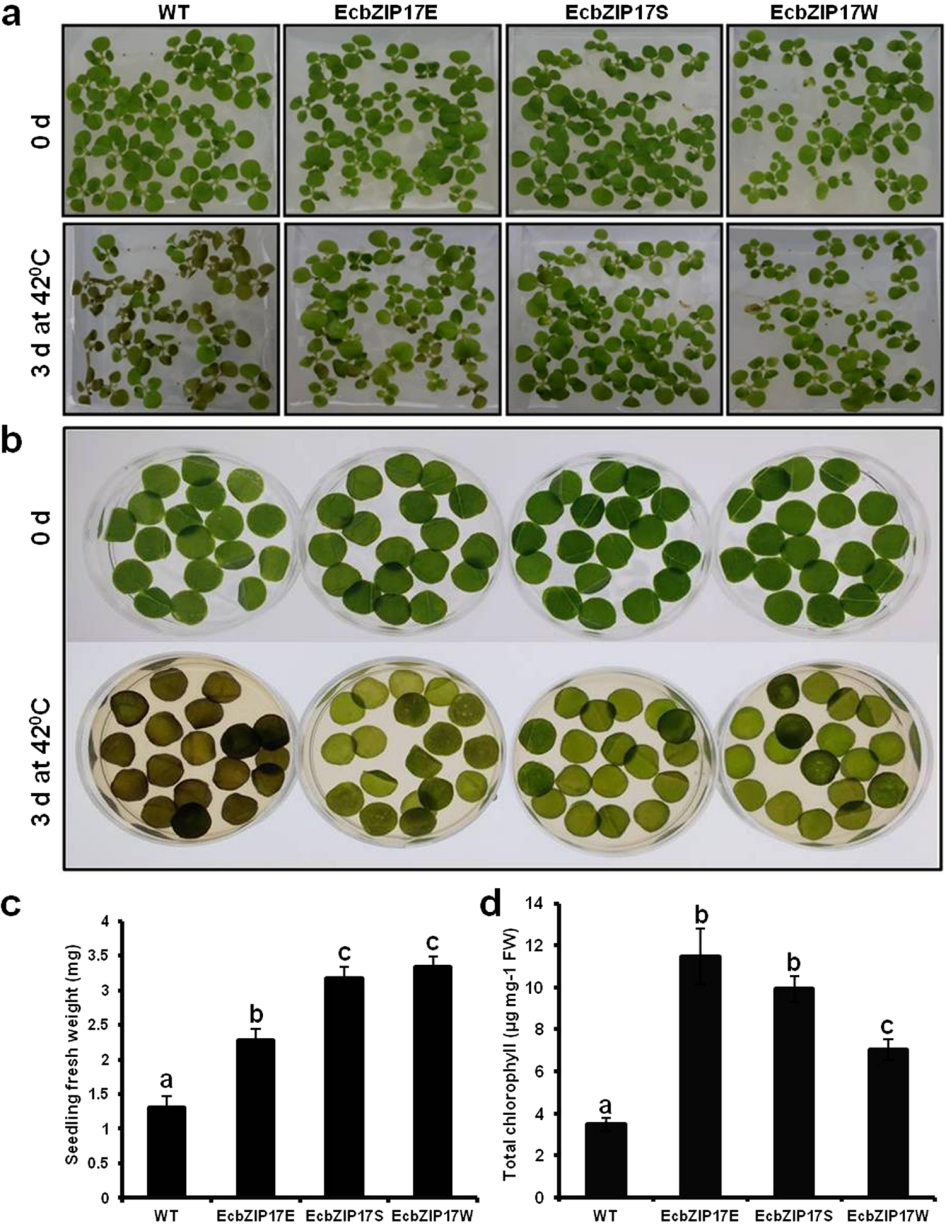
*EcbZIP17* over-expressing tobacco plants tolerant to heat stress. (**a**) 20 d old WT and *EcbZIP17*-T1 transgenic seedlings (EcbZIP17E, EcbZIP17S, EcbZIP17W) were germinated on MS medium and subjected to continuous heat stress at 42 °C and images were captured after three days. (**b**) Leaf discs from WT and transgenic lines were prepared and subjected to continuous heat stress at 42 °C and images were captured after three days. (**c**) Seedling fresh weight and (**d**) total chlorophyll content after three days of heat stress. According to one way ANOVA values of transgenic plants significantly different from WT and indicated by lower case letters above the error bars (P < 0.05).

Under heat stress of whole seedlings, the leaf colour of WT plants became watery brown, and plants started dying after 3 d, whereas the leaves of transgenic plants retained their green colour, and the plants survived (Fig. 6a). The fresh weight of transgenic seedlings was significantly increased after 3 d of treatment (Fig. 6c). In leaf disc assay, after the heat treatment, discs of transgenic plants remained green in colour, whereas those of WT plants turned watery brown (Fig. 6b). A significant retention of the chlorophyll content was observed in transgenic plants compared with WT plants (Fig. 6d).

### Tolerance of *EcbZIP17* transgenic plants to DTT stress

**Figure 7:**
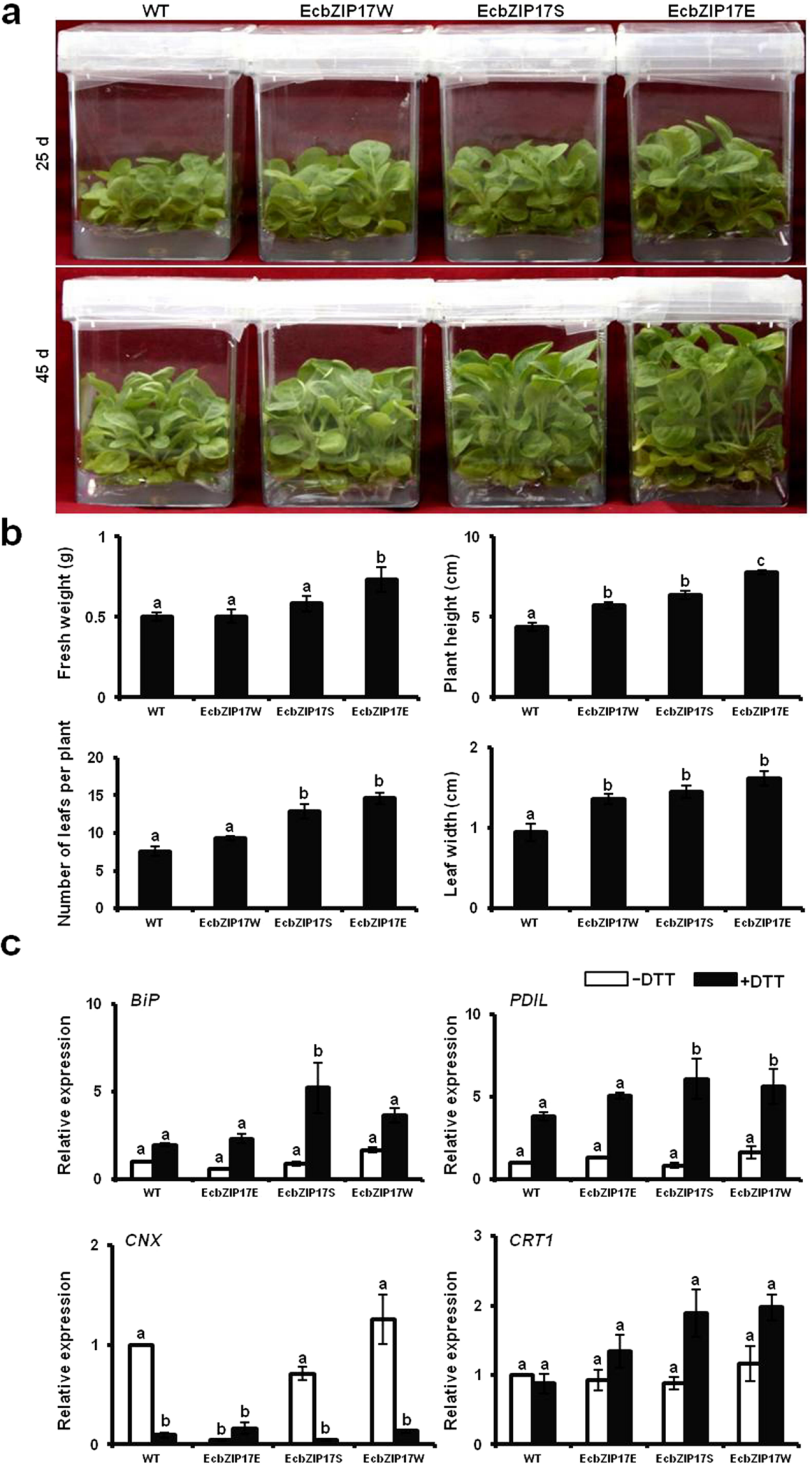
*EcbZIP17* over-expressing tobacco plants tolerant to DTT stress. (**a**) WT and EcbZIP17-T1 transgenic lines (EcbZIP17E, EcbZIP17S, EcbZIP17W) were germinated on MS plain and MS medium supplemented with Kanamycin (100 mg/l), respectively, and ten days after germination, fifteen seedlings from each line were placed in magenta boxes having MS medium supplemented with 2.5 mM DTT and images were captured after 25 and 45 d. (**b**) Fresh weight, plant height, number of leaves per plant and leaf width was measured after 45 d. (**c**) Fifteen days old seedlings of WT and EcbZIP17-T1 transgenic lines (EcbZIP17E, EcbZIP17S) treated with 2.5 mM DTT for 6 h. Expression levels of ER stress responsive genes *Bip*, *PDIL*, *CNX* and *CRT1* were detected by qRT-PCR. L25 was used as reference gene and the data was normalized to WT under control condition. Data represents mean ± SE of atleast five replicates. According to one way ANOVA (P < 0.05), different lower case letters above the error bars indicate significant difference after normalization.

Under 2.5 mM DTT stress, morphological changes in transgenic and WT plants were observed after 25 d and 45 d of treatment (Fig. 7a). Transgenic plants showed a significant increase in plant height, number of leaves per plant, average diameter of leaf, and fresh weight compared with WT plants (Fig. 7b). Under 2.5 mM and 3 mM DTT stress, the surface area of leaves of transgenic plants was larger compared with that of WT plants (Supplementary Fig. 9a,b). Under 3 mM DTT stress, the vegetative growth of EcbZIP17S was less affected compared with that of WT plants (Supplementary Fig. 9c), whereas under 4 mM DTT stress, all WT plants lost their chlorophyll content and became bleached after 30 d of treatment, whereas transgenic plants retained their chlorophyll content (Supplementary Fig. 9d). The findings of the DTT experiment suggested the significant role of *EcbZIP17* in UPR stress tolerance. To further confirm the role of *EcbZIP17* in ER stress at the molecular level, the UPR stress responsive genes *BiP*, *PDIL*, *CNX*, and *CRT1* were used for expression analysis. Under optimal growth conditions, all the genes showed almost similar expression in transgenic and WT plants, whereas after 6 h of 2.5 mM DTT stress, all the genes were upregulated in transgenic plants compared with WT plants (Fig. 7c), showing that the UPR pathway in transgenic plants was activated under stress conditions.

## Discussion

Plants are exposed to different biotic and abiotic stresses during their lifespan and hence, have developed mechanisms to cope with the adverse environmental conditions. Plant species native to harsh environmental conditions and the wild relatives of crop plants are considered rich reservoirs of stress tolerance genes. However, crop plants lost many of the stress tolerant alleles due to domestication and breeding. To improve crop growth and yield under stress conditions, many genes have been characterised at the molecular level, but most of them only provide tolerance to one or a few stresses. In the present study, we identified and characterised a gene that can provide tolerance to multiple abiotic stresses and can perform better under optimal growth conditions.

*EcbZIP17* was isolated from finger millet, a crop plant that is tolerant to harsh environmental conditions^50^. This gene encodes a type II transmembrane protein category and thus, contains NLS, TMD, S1P, and S2P sites, which are characteristic of MTTFs. In *Arabidopsis*, there are four type II MTTF members, *AtbZIP17*, *AtbZIP28*, *AtbZIP49*, and *AtbZIP60*, which are ER stress signal transducers^17,25,26,51^. Of these, *AtbZIP17* and *AtbZIP28* are activated by regulated intramembrane proteolysis (RIP). *EcbZIP17* was isolated from a cDNA library of stressed finger millet plants and found to be upregulated under different abiotic stresses. Additionally, we characterised *EcbZIP17* under optimal growth conditions at different stages of plant development in order to better understand the role of inactivated bZIP17 in the ER membrane to answer the questions raised by Howell^24^. In this regard, transgenic tobacco plants overexpressing *EcbZIP17* were evaluated both under optimal growth conditions and different stress conditions.

Under optimal conditions, *EcbZIP17* transgenic plants showed better growth, including increased plant height, number of leaves, shoot girth, internode distance, and higher pod and seed yield, than WT plants, suggesting the role of *EcbZIP17* in vegetative growth and maturation. Previous studies revealed that overexpression of all MTTFs, *AtbZIP28*Δ, *AtbZIP60*, and *AtbZIP17*Δ from *Arabidopsis* and *EcbZIP60* from finger millet, negatively affected plant growth under optimal conditions, but improved tolerance under stress conditions^25,26,47^. *Arabidopsis* plants overexpressing *ZmbZIP17* showed normal growth under optimal conditions, similar to that of WT plants^52^. In the present study, transgenic plants showed a better vegetative growth under optimal conditions compared with WT plants, revealing the positive effect of overexpressing *EcbZIP17*. Although, the expression of ER stress responsive pathway genes, such as *BiP*, *PDIL*, *CNX*, and *CRT1*, in transgenic plants was similar to WT plants, the expression of brassinosteroid pathway genes such as expansin *NtEXP10* and auxin responsive gene *NtIAA14* showed upregulation at both seedling and flowering stage in transgenic plants compared to WT under optimal growth conditions.

Under all abiotic stresses *EcbZIP17* was upregulated in finger millet and also provided tolerance to drought, salt, heat, PEG 6000, and mannitol stress as well as improved growth in transgenic tobacco plants overexpressing the gene. Under different abiotic stress conditions, the growth parameters of transgenic plants, including biomass, leaf size, primary and secondary root length, and recovery after stress treatment, were significantly improved compared with those of WT plants. These results showed that *EcbZIP17* plays a key role in improving tolerance to multiple abiotic stresses. Previous studies reported that the overexpression of *AtbZIP60* and *AtbZIP28* in *Arabidopsis* provided tolerance to salt stress and heat stress, respectively^53,54^, whereas the overexpression of *EcbZIP60* in tobacco provided tolerance to both salt and drought stress^47^. Many other members of bZIP family TFs also have been reported to provide tolerance to multiple stresses.

*EcbZIP17* transgenic plants showed improved vegetative growth and leaf diameter under DTT stress. DTT is a reducing agent that disrupts the redox environment in the ER, which is required for the disulphide bridge formation in proteins, and results in the accumulation of unfolded proteins, triggering the ER stress response^55^. Abiotic stresses are known to interrupt protein folding and assembly in the ER and activate UPR^56^. The accumulation of unfolded proteins in the ER activates MTTFs, which are processed in the Golgi machinery and then, translocated to the nucleus, increasing the expression of UPR pathway genes such as *BiP*, *PDIL*, *CNX*, and *CRT1*^24,56^. In *Arabidopsis* plants transformed with chimeric constructs of *AtbZIP28Δ* and *AtbZIP28* with GFP, proteins were observed in ER structures; however, under tunicamycin and DTT stress, chimeric proteins were observed in the nucleus and triggered the activation of molecular chaperons that facilitated protein folding in the ER by preventing the disaggregation of proteins in the ER lumen^24,57^. Among the ER resident chaperons, BiP is the most well characterised^34,58^. The truncated form of AtbZIP28ΔC was shown to activate the promoter regions of BiP1 and BiP3, which were responsible for the ER stress response through the cis-elements P-UPRE and ERSE^59^. The overexpression of the truncated form of OsbZIP39ΔC and the ectopic expression of the ER stress transducer *ZmbZIP17* in *Arabidopsis* plants also significantly upregulated UPR pathway genes such as *BiP*, *PDIL*, *CNX* and *CRT1* under 2 mM DTT stress^52,60^. All these genes were also upregulated in *EcbZIP17* transgenic plants under DTT stress, confirming that stress tolerance was due to the same UPR pathway.

Under environmental stress conditions, EcbZIP17 was mobilised from the ER, processed by S1P and S2P in the Golgi apparatus, transported to the nucleus, and eventually activated UPR pathway genes. The DNA binding component of EcbZIP17 translocated to the nucleus after S2P intramembrane cleavage and functioned as a transcription factor, providing stress tolerance. Even in the absence of major stresses, small amounts of bZIPs translocate through the Golgi to the nucleus as plants are constantly subjected to various stresses of low intensity, such as fluctuations in temperature and irrigation times, which activate the brassinosteroid signalling pathway^61^. We also observed upregulation of brassinosteroid signalling pathway genes *EXP10*, *NtIAA14, NtSEB1, NtBZR1, NtNTR1 and NtPP2c4* in the transgenic lines at optimal growth conditions. Previous studies have shown that moderate water stress can stimulate primary root elongation and increase the rate of cell production^62^, whereas high temperature fluctuation can stimulate hypocotyl elongation^63^. These results might explain the improved growth of *EcbZIP17* transgenic plants under optimal growth conditions.

## Methods

### Growth conditions of finger millet plants

Seeds of the finger millet (*E. coracana L*. ‘MR1’) were germinated under culture room conditions (temperature of 25 ± 1 °C, relative humidity of 70–80%, light intensity of 100 μmol m^−2^ s^−1^, and 16-h light/8-h dark photoperiod), and 7-d-old seedlings were used for different stress treatments^48^.

### Isolation and sequence analysis of *EcbZIP17*

We generated EST data from heat-stressed finger millet seedlings, and selected *EcbZIP17* for further characterisation due to its relative higher expression. To obtain the full-length sequence 5′ RACE was performed using heat-stressed RNA sample. A cDNA library was prepared using SMART RACE cDNA amplification kit (Clontech, USA), and a series of nested PCR reactions was performed. Amplicons obtained from nested PCRs were cloned using the pGEMT easy vector (Promega, USA), and at least four colonies were sequenced and submitted to NCBI (Accession no. KF245640.1). The full-length *EcbZIP17* cDNA was cloned incorporating *Kpn*I and *Sal*I restriction sites in the forward and reverse primers, respectively.

### *In silico* analysis of *EcbZIP17*

For nucleotide and protein sequence analysis, the National Centre for Biotechnology Information (NCBI; http://www.ncbi.nlm.nih.gov/) was used. The basic leucine zipper (BRLZ) and transmembrane domains in EcbZIP17 were observed using the Simple Modular Architecture Research Tool (SMART; http://smart.embl-heidelberg.de/). The leucine rich nuclear export signals (NES) were predicted by the NetNES 1.1 server (http://www.cbs.dtu.dk/services/NetNES/), whereas the nuclear localisation signal was predicted by the cNLS Mapper tool (http://nls-mapper.iab.keio.ac.jp/cgi-bin/NLS_Mapper_form.cgi)^2^. The CELLO 2.5 Sub-Cellular Localisation predictor (http://cello.life.nctu.edu.tw/) was used for subcellular localisation. The alignment analysis of EcbZIP17 and its homologs obtained from related species was carried out using CLUSTALW2 (http://www.ebi.ac.uk/Tools/msa/clustalw2/), and the phylogenetic tree was constructed using MEGA 5.0. The Multiple EM for Motif Elicitation software (MEME; http://meme-suite.org/tools/meme) was used for identifying motifs present in CLUSTALW2 aligned sequences.

### Expression analysis of *EcbZIP17* in different plant tissues and under different stress conditions

Finger millet seedlings at the three-leaf growth stage were subjected to different abiotic stressors, including heat at 42 °C, dehydration by removing seedlings from the medium and keeping them on 3-mm Whatman sheet, sodium chloride (NaCl; 250 mM), mannitol (300 mM), abscisic acid (ABA; 100 µM), hydrogen peroxide (H_2_O_2_; 25 mM), and DTT (2.5 mM)^47,64,65^. Seedling samples were collected after 2 h, 4 h, 6 h, 8 h, and 24 h of treatment. Total RNA was isolated from whole seedlings as well from leaf, shoot, root, and panicle samples of matured plants using Spectrum plant total RNA isolation kit (Sigma, USA) to study the expression pattern of *EcbZIP17*. 1 μg of total RNA was reverse transcribed to single-stranded cDNA using Affynityscript qRT-PCR cDNA synthesis kit (Stratagene, USA). A 1:5 dilution of cDNA pool was used as a template for qRT-PCR expression analysis with gene-specific primers. The qRT-PCR conditions were as follows: 95 °C for 5 min, followed by 38 cycles of 95 °C for 5s, 58 °C for 10s and 72 °C for 25s. The housekeeping gene β-tubulin was used for qRT-PCR normalisation^66,67^.

### Binary vector construction

A pGEMT-*EcbZIP17* plasmid confirmed by sequencing was restricted with *Kpn*I and *Sal*I restriction enzymes. The pCAMBIA2300 binary vector with CAMV35S enhancer-promoter and *Npt*II selection marker was linearized by same enzymes. The restricted ORF of *EcbZIP17* was ligated to the linearized pCAMBIA2300 vector at 4 °C overnight using T4 DNA ligase (Fermentas, USA). The resulting construct was mobilised into *Agrobacterium tumefaciens* EHA105 competent cells by the freeze-thaw method^68^.

### Development of transgenic tobacco plants

Leaves of 20-d-old healthy tobacco (*Nicotiana tabacum* L. ‘Petit Havana’) plants were used for *Agrobacterium*-mediated genetic transformation. Leaf discs were cut and incubated in Murashige and Skoog (MS) pre-culture medium supplemented with 2.5 mg L^−1^ 6-benzylaminopurine (BAP) and 0.1 mg L^−1^ 1-naphthaleneacetic acid (NAA). After 2 d, leaf discs were infected by *A. tumefaciens* with the pCAMBIA2300-*EcbZIP17* vector for 20 min, placed on filter paper to optimise the density of *A. tumefaciens* cells, and cultured in MS co-culture medium supplemented with 2.5 mg L^−1^ BAP and 0.1 mg L^−1^ NAA under dark conditions for 3 d. Next, the leaf disks were transferred to MS selection medium supplemented with 2.5 mg L^−1^ BAP, 0.1 mg L^−1^ NAA, 200 mg L^−1^ kanamycin, and 500 mg L^−1^ cefotaxime and cultured under light conditions. After 15 d, leaf discs were transferred to fresh selection medium. During this process, calli developed from the infected areas of leaf discs, and the newly developed shoots from calli were sub-cultured to fresh medium in bottles. The elongated shoots were shifted to MS rooting medium supplemented with 200 mg L^−1^ kanamycin and 500 mg L^−1^ cefotaxime^69^. The putative transgenic plants were then transferred to small pots, containing soilrite, and later acclimatised to a glasshouse.

### Confirmation of putative transgenic plants

Genomic DNA from transgenic and wild type (WT) plants was isolated using the CTAB (N-cetyl-N,N,N-trimethyl ammonium bromide) method, and the quality and quantity were checked using Nanodrop (Thermo Scientific, USA) and 0.8% agarose gel electrophoresis. The presence of T-DNA was carried out by PCR with gene specific and *Npt*II specific primers and by Southern blot hybridisation.

### Southern blot hybridisation

Southern blot hybridisation was carried out to identify the copy number and stable integration of *npt*II in the genome of *EcbZIP17* transgenic plants. A total of 20 μg of genomic DNA from *EcbZIP17* transgenic and WT plants was digested by *Hind*III enzyme. The restricted genomic fragments were resolved by 0.8% agarose gel electrophoresis and transferred to a nitrocellulose membrane (Hybond-N+; Amersham Pharmacia, UK) via the capillary method. The probe preparation was performed using *npt*II. A 750-bp fragment of the gene was PCR amplified, gel purified, and α[32 P]-dCTP was labelled using the mega prime DNA labelling system (Amersham Biosciences, UK). Further hybridisation and washing were carried out at 65 °C. The hybridised membrane was exposed to an X-ray film in an intensifying cassette under dark room conditions. The cassette was placed at –80 °C for 3 d and developed to visualise the bands^65^.

### Expression analysis of *EcbZIP17* transgenic plants

qRT-PCR was performed to check the levels of mRNA in transgenic plants overexpressing *EcbZIP17*. Total RNA was isolated from transgenic and WT plants, and cDNA was prepared using 1.0 μg of total RNA. The expression analysis of *EcbZIP17* was performed by qRT-PCR, considering the Ct value of the lowest expressing plant as 1-fold for comparison. The housekeeping gene L25 was used for qRT-PCR normalisation^70^.

### Analysis of *EcbZIP17* transgenic plants under stress treatments

Confirmed T1 seedlings obtained from independent T0 plants were analysed under various abiotic stresses. All the experiments were carried out in three replicates. After sterilisation, *EcbZIP17*-T1 transgenic plants were germinated on MS medium supplemented with 100 mg L^−1^ kanamycin, whereas WT seeds were germinated on MS medium without kanamycin. At 10d after germination, seedlings were placed on MS medium supplemented with different abiotic stressors, including 10% PEG 6000, 400 mM mannitol, and MS medium supplemented with endoplasmic reticulum (ER) stressor^52,71^ (2.5 mM, 3 mM, and 4 mM DTT). To analyse the salinity tolerance at germination level, seeds of *EcbZIP17* transgenic plants and WT were germinated on MS agar medium supplemented with 250 mM NaCl^2^ and to analyse salt tolerance at mature plant level, 30-d-old transgenic and WT plants were saturated in 200 mM NaCl solution at 3-d intervals continuously for one month. To evaluate response to drought stress at mature plant level, transgenic and WT plants were subjected to water withdrawal for 11 d. Further for recovery study from drought stress, the plants were saturated with water^65^. For the heat stress treatment, 20-d old transgenic plants were grown on MS medium without kanamycin at 42 °C for 3d. Additionally, a leaf disc assay was carried out using mature leaves, keeping 18 leaf discs of transgenic and WT plants in petri dishes, containing autoclaved water at 42 °C for 3 d.

### Chlorophyll estimation and membrane stability index (MSI)

Total chlorophyll was estimated from 100 mg of leaf tissue incubated in 1:1 acetone: dimethyl sulfoxide solution overnight. The absorbance was measured at 663 nm, 645 nm, and 710 nm using a UV/Visible spectrophotometer (Optima, Japan). The total chlorophyll content was calculated as described by Mohanty & Boyar^72^. Total chlorophyll: (a + b) = (A645–A710) 20.2 + (A663–A710) 8.02 and expressed in µg ml^−1^.

The MSI of transgenic and WT plants was estimated as described by Sairam *et al*. using an electrolyte leakage conductivity meter^73^. To measure the electrical conductivity E1, samples were placed in test tubes containing 10 ml of deionised water at 42 °C for 30 min, whereas to measure the electrical conductivity E2, the same samples were placed in test tubes containing 10 ml of deionised water at 100 °C for 10 min. The MSI was calculated as follows:

$${\rm{MSI}}=1-{\rm{E}}1/{\rm{E}}\times 100.$$

### Statistical analysis

All experiments were carried out in three replicates. One-way analysis of variance (ANOVA) was performed to determine significant differences between the means within each treatment at *p* < 0.05. GraphPad Prism 5.0 software (Graph Pad, USA) was used for statistical analysis^74^. Lower case letters were used to indicate the significant difference above the error bars.

## Electronic supplementary material

Supplementary information
