# Supplementary material for: The membrane tethered transcription factor EcbZIP17 from finger millet promotes plant growth and enhances tolerance to abiotic stresses
Source: Sci Rep. 2018 Feb 1;8:2148. doi: 10.1038/s41598-018-19766-4 (PMC5794737; doi:10.1038/s41598-018-19766-4)
Supplement: Supplementary file 1 — Supplementary information [file 41598_2018_19766_MOESM1_ESM.doc]

**The membrane tethered transcription factor *EcbZIP17* from finger millet promotes plant growth and enhances tolerance to abiotic stresses**

Ramakrishna Chopperla^1, 2^, Sonam Singh^1^, Raghavendrarao Sangala^1^, Jasdeep C. Padaria^1^, Sasmita Mohanty^2^, Tilak Raj Sharma^1^ and Amolkumar U. Solanke^1^*

^1^ICAR-National Research Centre on Plant Biotechnology, Pusa Campus, New Delhi-110012

^2^School of Biotechnology, KIIT University, Bhubaneswar, Odisha-751024, India

*Email: amolsgene@gmail.com

**Author for correspondence:**

Amolkumar U. Solanke

Tel: +911125842789

Email: [amolsgene@gmail.com](mailto:amolsgene@gmail.com)

**Supplemental Figure 1. Full length sequence of EcbZIP17.** Nucleotide sequence of EcbZIP17 with its deduced amino acid sequence. Full length open reading frame (ORF) of EcbZIP17 cDNA sequence is 1722 bp. The 5’ and 3’ UTR regions (1-19 and 1722-1783) are depicted by red colour.

**
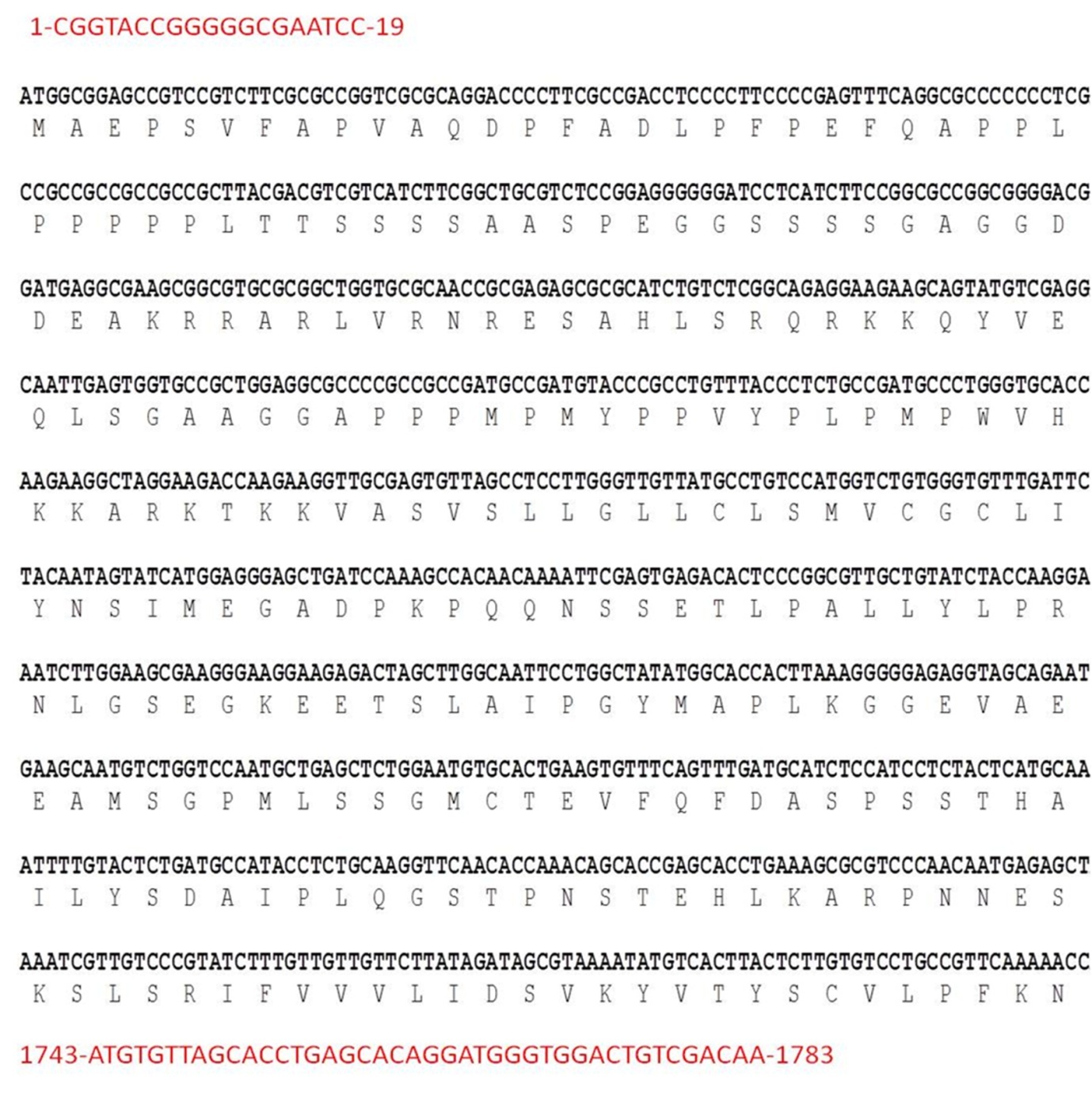
**

**Supplemental Figure 2. Prediction of subcelular localization, nuclear export signals and transmembrane regions in EcbZIP17 protein.** **(A)** Prediction of subcelular localization signal in EcbZIP17 protein through CELLO online tool. **(B)** Prediction of leucine rich nuclear export signals (NES) in EcbZIP17 protein through NetNES online tool. **
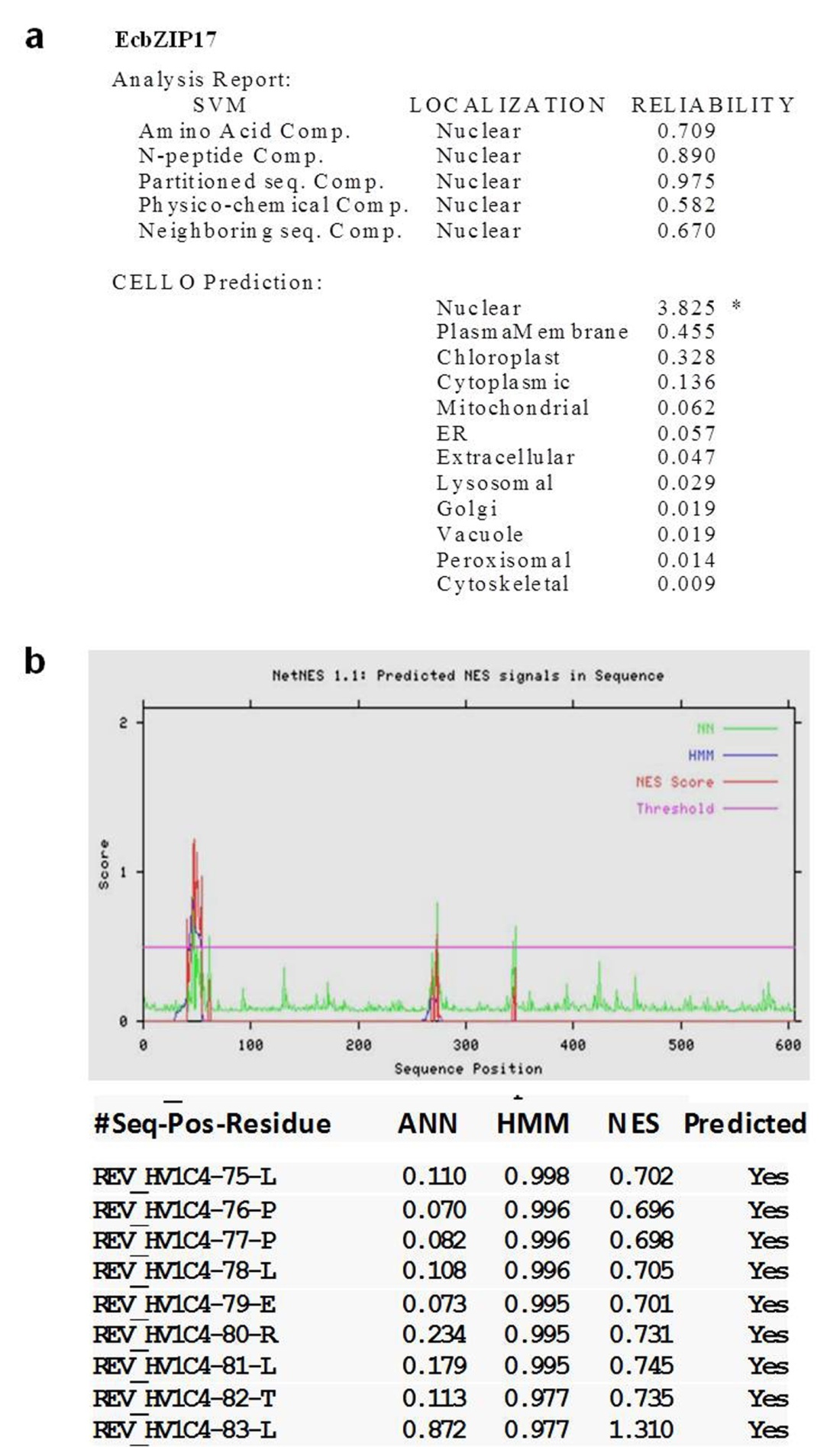
**

**Supplemental Figure 3. Bioinformatics analysis of EcbZIP17 protein: (A)** Prediction of major domain regions, Nuclear localization signal, and S1P protease site in EcbZIP17 protein **(B)** Multiple sequence alignment of EcbZIP17 and other proteins **(C)** Phylogenetic tree for EcbZIP17 and other proteins **(D)** Prediction of putative motifs in EcbZIP17 and other proteins through MEME tool.

**
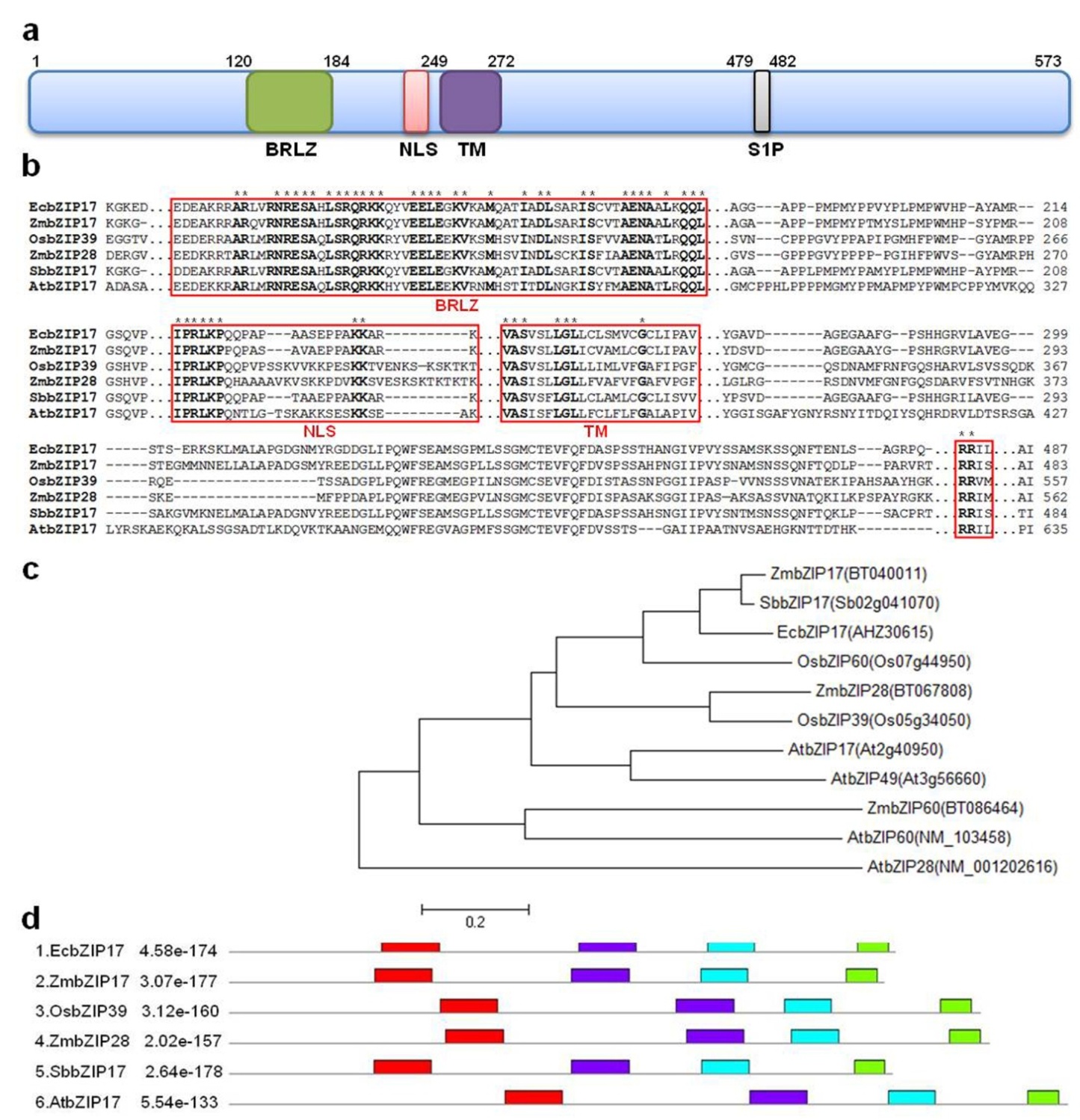
**

**Supplemental Figure 4. Schematic representation of T-DNA region carrying EcbZIP17 gene, southern and qPCR analysis of *EcbZIP17*-T1 transgenic lines.** **(A)** T-DNA region of binary vector pCAMBIA2300 carrying EcbZIP17 gene under 35S enhancer promoter. *NPTII* (Neomycin Phosphotransferase) gene for Kanamycin resistance, RB-Right border, and LB-Left border. **(B)** Gene specific (937 bp) and *nptII* (750 bp) specific PCR conformation of EcbZIP17-T1 transgenic Tobacco lines. **(C)** Southern hybridization analysis of EcbZIP17-T1 transgenic lines using *NptII* probe. 20 µg genomic DNA from transgenic lines and wild type were digested with the *HindIII* restriction enzyme. *EcbZIP17*B to *EcbZIP17*Z indicates different events of transgenic lines, WT indicates wild type. The size of the DNA in Kb indicated at left. **(D)** Expression analysis of *EcbZIP17* transcript in different T1 transgenic lines through qRT-PCR.

**
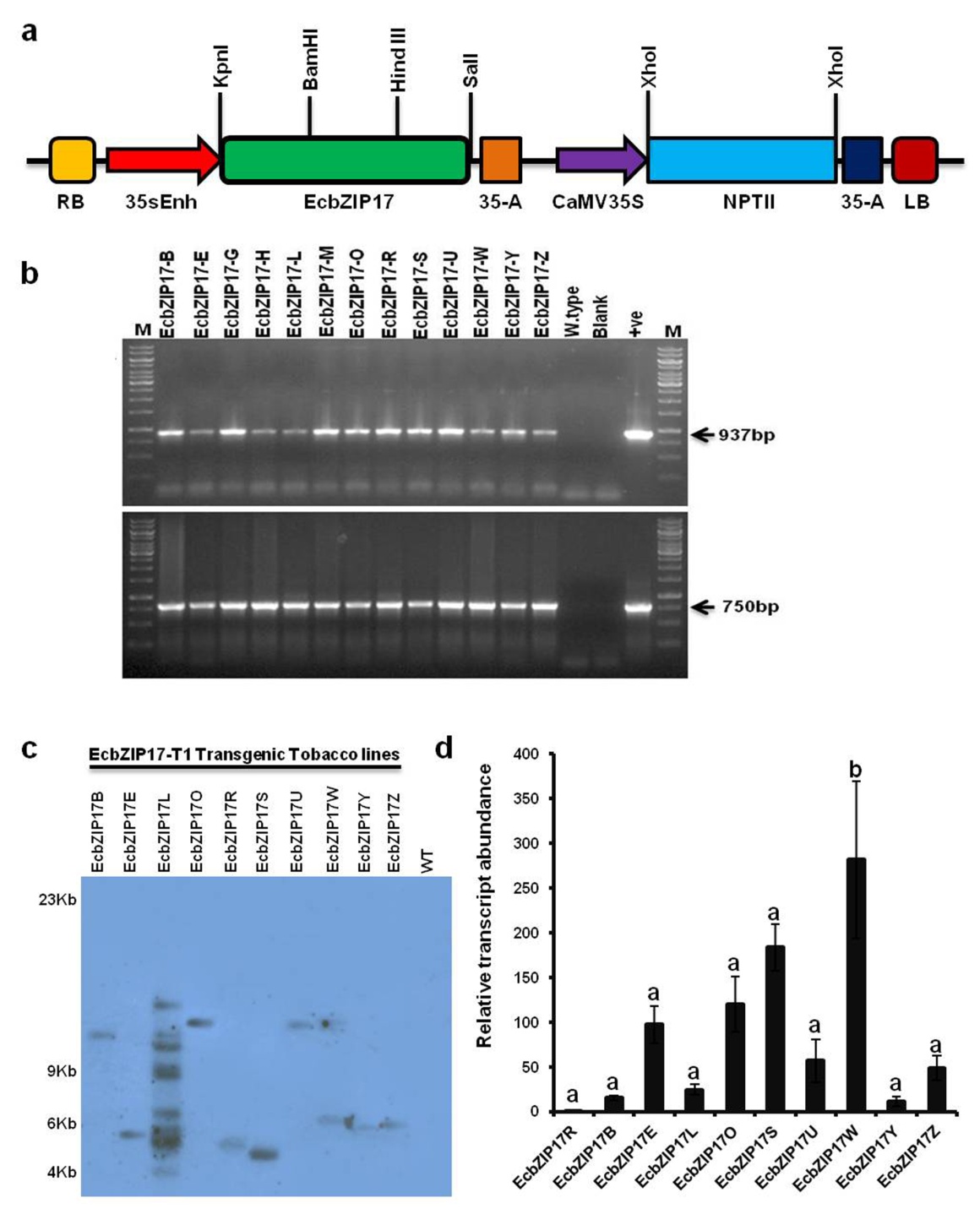
**

**Supplemental Figure 5. Membrane stability index (MSI).** Graph chart representation of Membrane stability index for WT and EcbZIP17-T1 transgenic lines subjected to 250 mM NaCl stress.

**
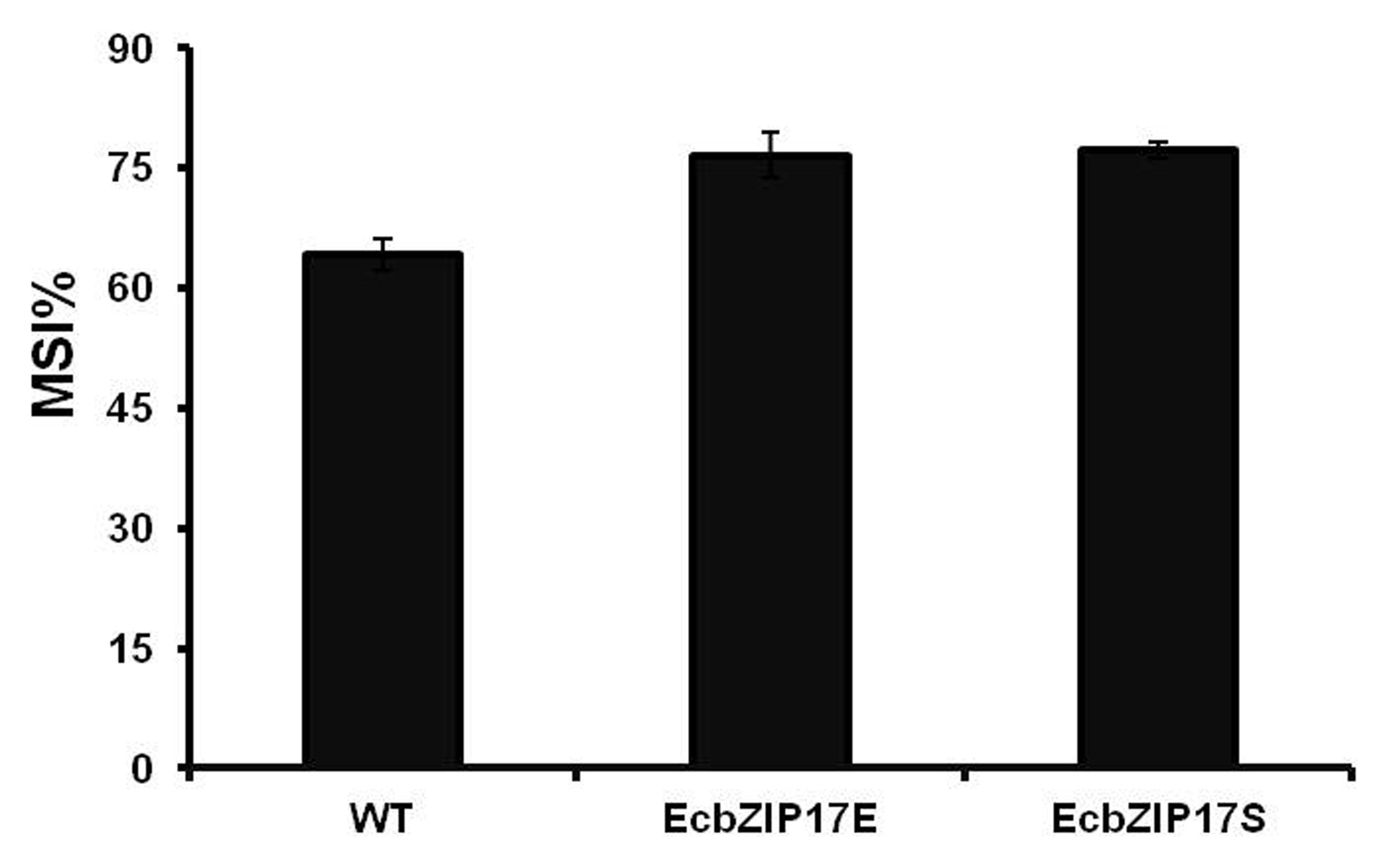
**

**Supplemental Figure 6. Mannitol stress tolerance of EcbZIP17-T1 transgenic lines.** 10 d-old seedlings of WT and EcbZIP17-T1 transgenic lines were placed on MS agar medium supplemented with 400mM Mannitol and the photographs were taken after 15, 25 and 45 d, respectively.


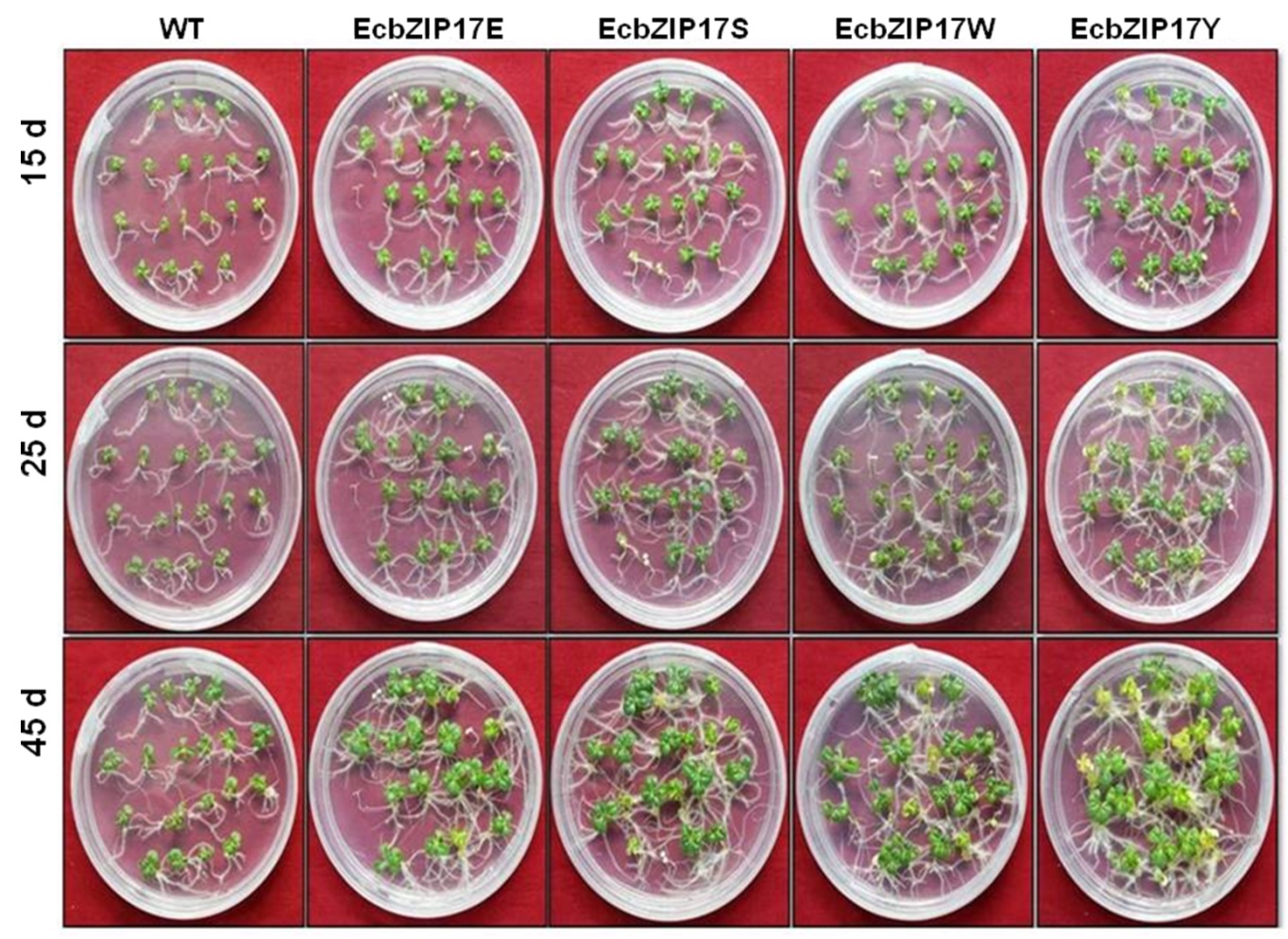


**Supplemental Figure 7. 10%PEG tolerance of EcbZIP17-T1 transgenic lines.** (a) Wild type and EcbZIP17-T1 transgenic lines (EcbZIP17E, EcbZIP17S, EcbZIP17W) were germinated in MS plain and MS with Kanamycin medium and after 10 days, 15 seedlings from each were transferred to MS medium supplemented with 10%PEG and photographs were taken after 15, 25 and 35 days respectively. (b) Graph chart representation of fresh weight and dry weight of wild type and transgenic lines after 35 days of 10% PEG (6000) stress.

**
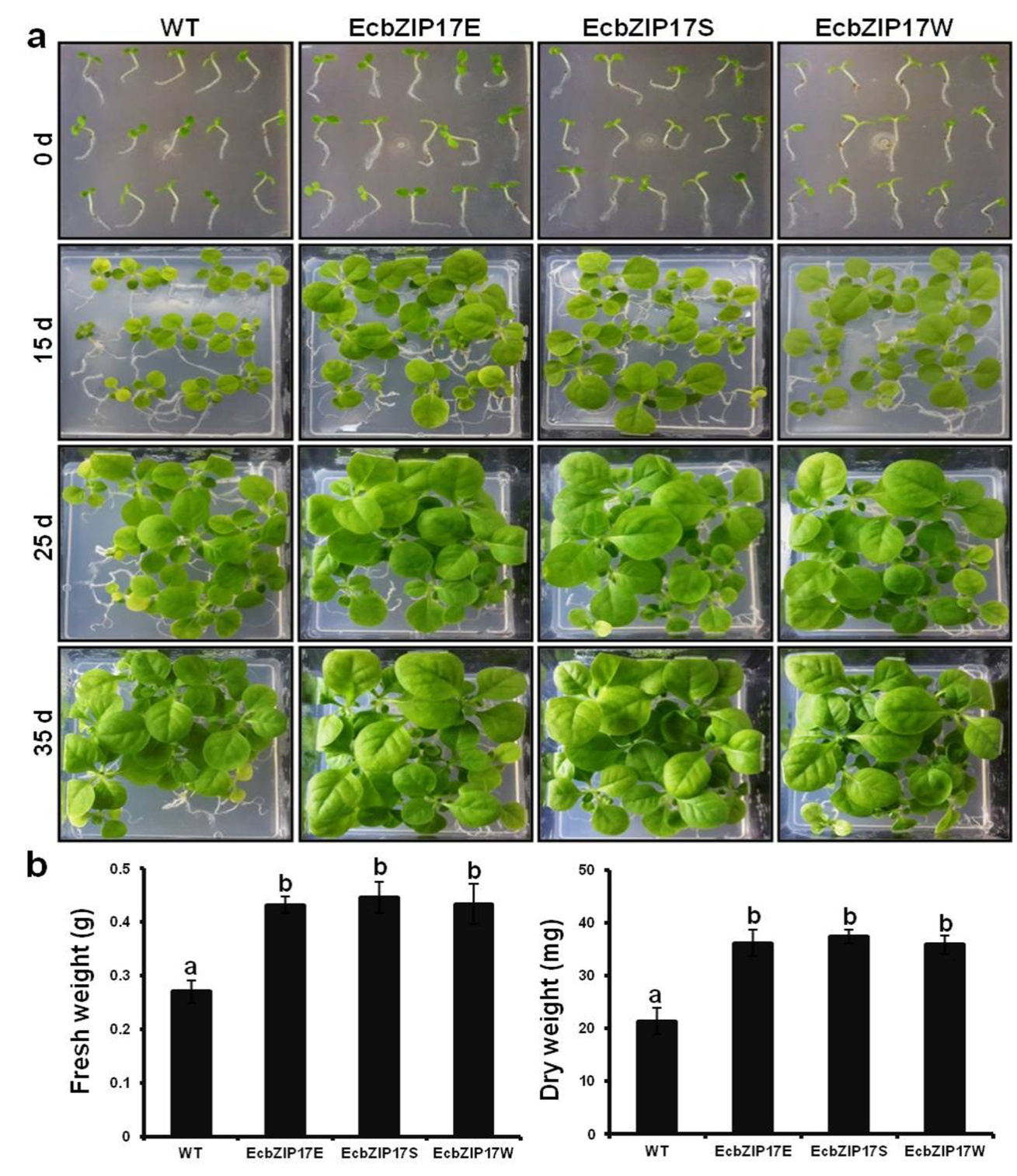
**

**Supplemental Figure 8. Chlorophyll content.**  Graph chart representation of Chlorophyll content for WT and EcbZIP17-T1 transgenic lines subjected to 10% PEG stress.

**
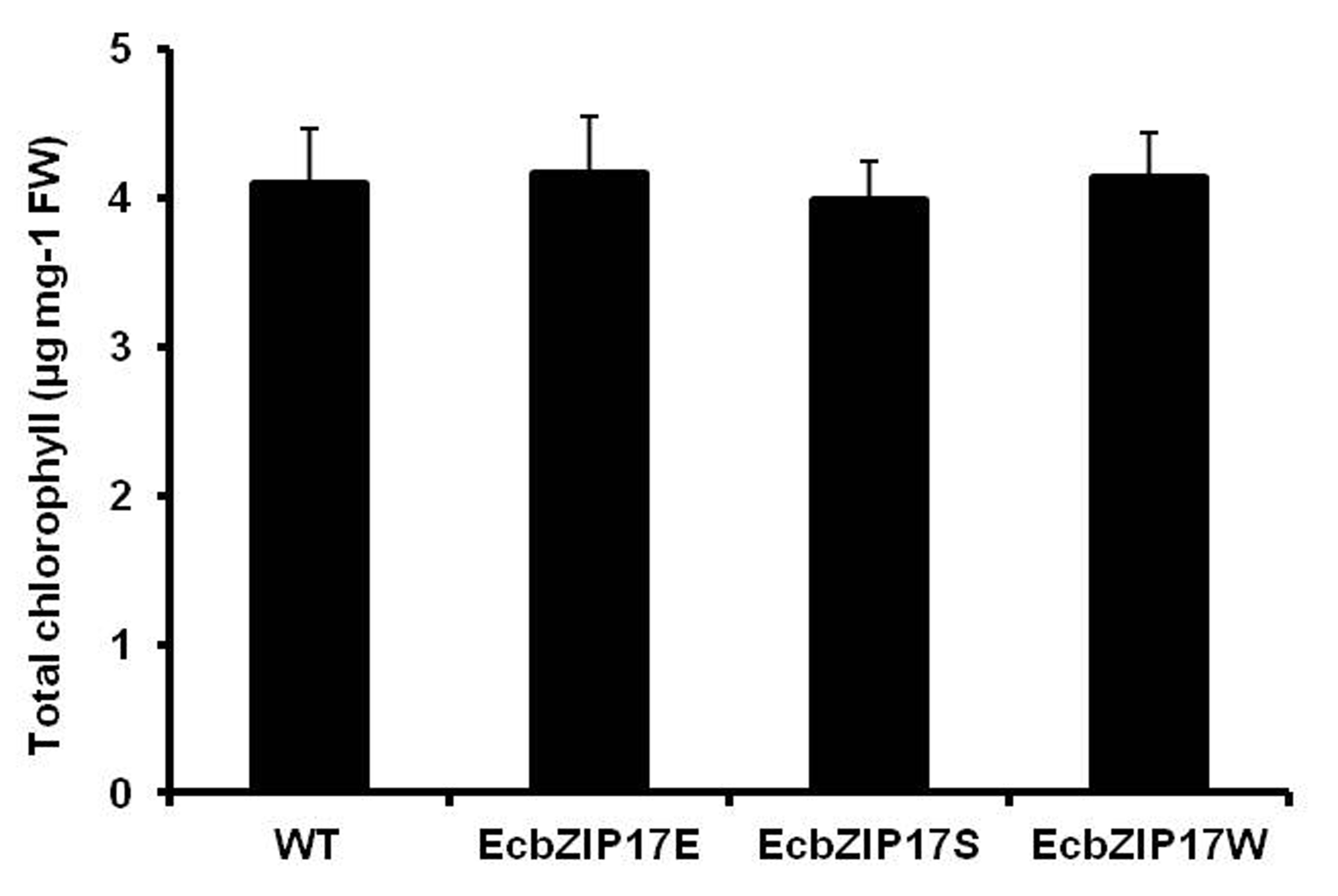
**

**Supplemental Figure 9. DTT stress tolerance of EcbZIP17-T1 transgenic lines.**  **(A)** Leafs collected from WT and EcbZIP17-T1 transgenic lines subjected to 2.5 mM, and **(B)** 3 mM DTT stress. WT and the EcbZIP17S transgenic seedlings were placed on MS medium supplemented with 3 mM DTT **(C),** and 4 mM DTT **(D)** and the photograph was taken after one moth time period. In 3 mM DTT, significant vegetative growth difference was observed in transgenic seedlings (EcbZIP17S) compared to WT, where as in 4 mM DTT, significant survivalence rate of transgenic line seedlings (EcbZIP17S) was observed compared to WT.

**
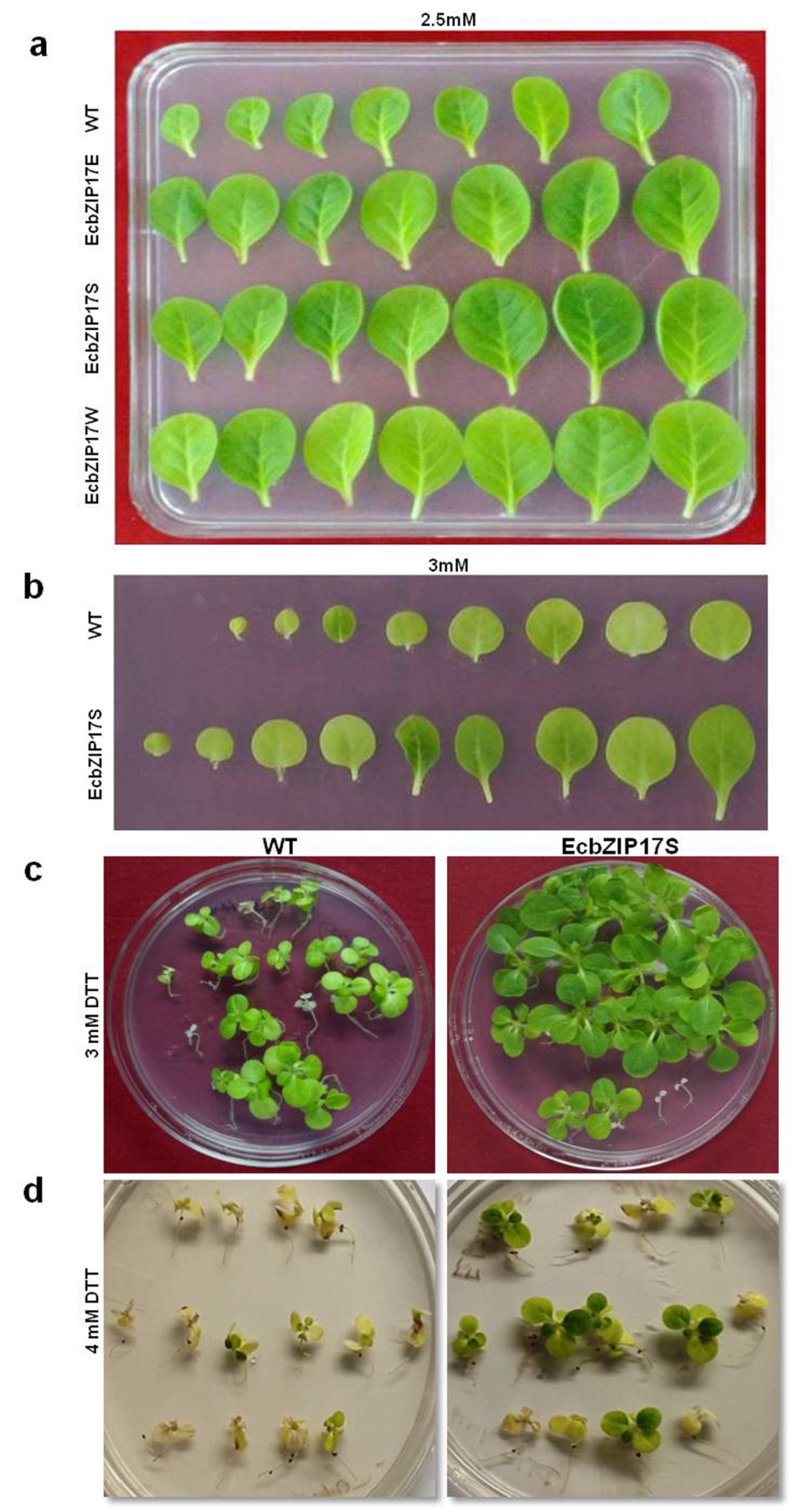
**

**Supplemental Table 1.**  **List of primers used in the study.**

| L25_F | 5' -CCCCTCACCACAGAGTCTGC-3' |
| --- | --- |
| L25_R | 5' -AAGGGTGTTGTTGTCCTCAATCTT-3' |
| βTubulin_F | 5' -GAGCAGTTCACAGCCATGTT-3' |
| βTubulin_R | 5' -CCAGATCGTTCATGTTGCTCTC-3' |
| EcbZIP17_NR1 | 5' -GATTTTGTACTCTGATGCCATGCCT-3' |
| EcbZIP17_NR2 | 5' -GTCTACTCCAGTGCCATGTCGAAGT-3' |
| EcbZIP17FL_F | 5' -CGGTACCGGGGGCGAATCCAT-3' |
| EcbZIP17FL_R | 5' -TTGTCGACAGTCCACCCATCCTGTGCTC-3' |
| EcbZIP17qRT_F | 5' -GTTGTCCCGTATCTTTGTTGTTG-3' |
| EcbZIP17qRT_R | 5' -TGCTCAGGTGCTAACACATTTA-3' |
| NtBiP1_F | 5' -GAACGTATGGTCAAGGAGGCCG-3' |
| NtBiP1_R | 5' -CTCAGCACTCTGGTTGTCGTCC-3' |
| NtCRT1 _F | 5' -CATTCCCAACCCGGAGTACAAG -3' |
| NtCRT1 _R | 5'-CTTGGCATACTCTGGATCGTC-3' |
| NtPDIL_F | 5' -GTGCTGTTGGAGTTCTATGCAC -3' |
| NtPDIL_R | 5' -GTGACAAGTTACCGGAGGCAG-3' |
| NtCNX _F | 5' -GTTCTTCACTTGAGGGACTATC -3' |
| NtCNX _R | 5' -GTAATTCCAGCCACTACACTCG-3' |
| NtExp10_F | 5' -CCTAGCTTTGGGACCCTATAATTC-3' |
| NtExp10_R | 5' -CACCACTGCAATCCTCTTCT-3' |
| NtIAA14_F | 5' -GAGATGTACCTTGGCAGATGTT-3' |
| NtIAA14_R | 5' -CTATTGCTCAGTTCCTGCTCTT-3' |
| NtSEB1_F | 5' -CGCGGTGCAATTGGATAAAG-3' |
| NtSEB1_R | 5' -GCCTTGCATGAACAAGGTATTG -3' |
| NtBZR1_F | 5' -GGAAGTGCTCGCGTATGATTA-3' |
| NtBZR1_R | 5' -ACTCGGACATCCACTGAAATG-3' |
| NtNRT1_F | 5' - GTTCCCATTTGTCGTCGTTTC -3' |
| NtNRT1_F | 5' -TTAGGGCAGCAGCAATCAT-3' |
| NtPP2c4_F | 5' -TACCACCACACACTTGGTTC-3' |
| NtPP2C4_F | 5' -CTCTACTTCTGAGACTGCTTCTTC-3' |
